# Outcomes of Beinaglutide on Weight Loss in Patients With Diabetes or Obesity: A Systematic Review and Meta-Analysis

**DOI:** 10.7759/cureus.100224

**Published:** 2025-12-27

**Authors:** Jimmy Wen, Alina Truong, Denise Nadora, Christiane How-Volkman, Ethan M Bernstein, Megan Kou, Arsh Alam, Jose Puglisi, Eldo Frezza

**Affiliations:** 1 Physical Medicine and Rehabilitation, California Northstate University College of Medicine, Elk Grove, USA; 2 Cardiology, California Northstate University College of Medicine, Elk Grove, USA; 3 Neurology, California Northstate University College of Medicine, Elk Grove, USA; 4 College of Medicine, California Northstate University College of Medicine, Elk Grove, USA; 5 Orthopedic Surgery, California Northstate University College of Medicine, Elk Grove, USA; 6 Neurobiology, Physiology, and Behavior, University of California, Davis, Davis, USA; 7 Biostatistics, California Northstate University College of Medicine, Elk Grove, USA; 8 Surgery, California Northstate University College of Medicine, Elk Grove, USA

**Keywords:** beinaglutide, diabetes, meta-analysis, obesity, weight loss

## Abstract

Beinaglutide, a new short-acting glucagon-like peptide-1 (GLP-1) receptor agonist, has been investigated for use in patients with type 2 diabetes mellitus (T2DM) and obesity. This systematic review aims to assess the effectiveness of beinaglutide for weight loss and evaluate its safety profile in patients with T2DM or obesity. A systematic review search following the guidelines of the Preferred Reporting Items for Systematic Reviews and Meta-Analyses (PRISMA) was performed across four databases for studies evaluating the weight loss effects of beinaglutide. A meta-analysis using SPSS program version 29 (IBM Corp., Armonk, NY) was conducted to analyze weight loss effects in T2DM or obesity populations.

Nine studies (six randomized controlled trials, two prospective cohort studies, and one retrospective cohort study), comparing beinaglutide with other therapies such as metformin, insulin glargine, and lifestyle modifications, were included. A total of 1268 patients, with a mean age of 41.7 years (range: 25.4-53.3 years) and a mean follow-up of 47.6 weeks (range: 12-168 weeks), were included in this study. Meta-analysis for patients with obesity or T2DM showed weight reductions of 3.26 kg (95% CI: −4.03 to −2.49) and 6.52 kg (95% CI: −9.32 to −3.72), respectively. Adverse events (AEs) were observed in 382 (94.6%) participants receiving beinaglutide treatment (five studies). Beinaglutide demonstrated greater weight loss compared to placebo or active comparators but was associated with higher rates of AEs. Further long-term and comparative studies are needed to clarify its safety profile and potential advantages over current treatments.

## Introduction and background

Obesity, defined as a BMI ≥ 30, and overweight, defined as a BMI of 25-29.9, are among the most prevalent chronic health conditions in the United States. Data from a cross-sectional, nationally representative survey reported that nearly 73.8% of the US population is overweight and 42.8% of adults are obese [1]. Excess weight can lead to an increased risk of developing many health problems, such as type 2 diabetes mellitus (T2DM), hypertension, heart disease, stroke, and cancer [2]. However, despite the availability of lifestyle and pharmaceutical options, obesity and its metabolic sequelae remain uncontrolled in many patients, highlighting the need for more efficacious and accessible treatments [2].

Glucagon-like peptide-1 receptor agonists (GLP-1 RAs) are a class of medications used to treat T2DM and obesity. GLP-1 RAs promote weight loss by delaying gastric emptying, increasing insulin secretion while inhibiting glucagon release, and enhancing satiation, ultimately resulting in weight loss [3]. Based on their pharmacokinetic properties, GLP-1 agonists can be categorized into short-acting and long-acting agents [4]. Native GLP-1 is commonly degraded by dipeptidyl peptidase-4 (DPP-4) and neutral endopeptidase (NEP), lending to GLP-1’s short half-life of two minutes [5]. Short-acting GLP-1 agonists, such as exenatide and lixisenatide, have slightly longer half-lives of two to three hours and are administered twice daily or once daily, respectively. The difference in the amino acid sequence between native GLP-1 and currently available short-acting GLP-1 agonists is primarily due to N-terminal end variations that prevent degradation by DPP-4 [4]. Meanwhile, long-acting agents, such as semaglutide, dulaglutide, and exenatide microspheres, possess half-lives ranging from 13 hours to more than one week [4,6]. Similar to short-acting agents, long-acting agents exhibit increased resistance to DPP-4 degradation via amino acid substitutions, which ultimately translates through their prolonged half-lives [7].

Beinaglutide is a novel, short-acting GLP-1 agonist and is unique in that it shares 100% homology with endogenous GLP-1 [8,9]. Thus, unlike other GLP-1 RAs that use structural modifications to combat enzymatic degradation, beinaglutide exerts effects identical to physiological GLP-1, promoting weight loss through appetite suppression and delayed gastric emptying [8]. In mice studies, beinaglutide has also been shown to reduce fat mass and plasma lipid levels while improving insulin sensitivity in white adipose tissue and changing gene expression of various lipid classes [8]. Currently, beinaglutide is not approved by the US Food and Drug Administration (FDA) but was approved by the China Food and Drug Administration in December 2016 for the treatment of T2DM [10]. It has been increasingly used in real-world practice due to its short-acting nature and possible cost advantages compared to established GLP-1 RAs [10].

However, its clinical outcomes, efficacy, and safety profile have not been comprehensively examined in a meta-analysis. Several knowledge gaps exist yet to be explored, including its effectiveness in different patient populations (patients with diabetes versus non-diabetes) and existing evidence that is scattered across smaller clinical trials and observational studies. These studies are also limited by small sample sizes, variable dosing protocols, and heterogeneous comparators [9,10]. This systematic review and meta-analysis aims to assess the effectiveness of beinaglutide in promoting weight loss and evaluate its safety profile among patients with T2DM or obesity. We hypothesize that beinaglutide will produce substantial weight loss with low rates of adverse events (AEs)​​​​​​​.

## Review

Methods

Search Strategy and Information Sources

In June 2024, a systematic search adhering to the Preferred Reporting Items for Systematic Reviews and Meta-Analyses (PRISMA) guidelines was performed across the following databases: PubMed, Scopus, Embase, and the Cochrane Library. The same query was performed across all databases without modification, using the following Boolean search phrase: (beinaglutide) AND (weight loss OR outcomes OR efficacy OR diabetes OR diabetic OR overweight OR obesity) (Appendix 1). No restrictions were set on the search. However, gray literature, clinical trial registries, and non-English-language databases were not searched but are acknowledged as potential sources of unpublished or regional data.

Eligibility Criteria

The search strategy was guided using the PICOT (patient, intervention, comparison, outcome, time) method. The patient population included patients with obesity or T2DM over the age of 18 taking beinaglutide. The intervention and comparator were the use of beinaglutide alone or compared with a placebo or active comparator. Outcomes consisted of weight loss parameters and AEs. No restriction was placed on the mean follow-up time.

Studies were included if they were randomized controlled trials, comparative studies, or retrospective or prospective cohort studies investigating the effect of beinaglutide on weight loss. Studies were excluded if they met any of the following criteria: case reports, review articles, animal studies, cadaveric studies, expert opinions, abstracts, commentaries, non-English studies, or studies for which the full text was unavailable. The full study protocol was registered and can be referenced as PROSPERO: CRD42024562493.

Selection Process

Two authors independently reviewed each study using the predetermined eligibility criteria during title/abstract and full-text screening. If there were discrepancies with the decision, a rigorous re-review was performed until a consensus was reached. If consensus was not achieved, a third author was consulted to determine final inclusion or exclusion. All included articles underwent a thorough reference search to find any additional studies that could be added to this systematic review.

Risk of Bias Assessment

The Methodologic Index for Nonrandomized Studies (MINORS) criteria for non-randomized controlled trials (RCTs) and the Cochrane Risk of Bias tool for RCTs were utilized to assess the risk of bias and methodological quality [11,12]. MINORS scores were as follows: 0 (not reported), 1 (reported but inadequate), or 2 (reported and adequate) with a maximum score of 24 for comparative studies (12 categories) or 16 for non-comparative (8 categories). Articles that scored 1 or 2 for seven or more sections (11 or more for comparative) were considered low risk of bias, 1 or 2 for five to six sections (9 to 10 for comparative) were considered moderate risk of bias, and a score of 1 or 2 for four categories or less (eight or less for comparative) were considered high risk of bias.

Cochrane’s tool assessed seven domains: sequence generation, allocation concealment, blinding of participants and personnel, blinding of outcome assessors, incomplete outcome data, selective outcome reporting, and other sources of bias. These were evaluated as “high,” “low,” or “unclear” risk of bias. The risk of bias and methodological quality were determined by two independent reviewers, with discrepancies being reviewed until a consensus was achieved.

Data Extraction and Data Items

This systematic review included the following study variables: title, author, publication date, study year, number of patients, dosage of drug, mean age, mean follow-up time, BMI, weight loss outcomes, and AEs. Study variables such as weight loss outcomes and AEs were extracted as reported in the included studies, including the type and frequency as available. Grading or reclassification was not performed due to heterogeneity in reporting across studies. Descriptive statistics (such as means, percentages, and standard deviations) are included in this review when applicable and available. The data extraction database was compiled with Microsoft Excel (Microsoft Office, Version 16.80, 2023 (Microsoft Corp., Redmond, Washington)).

Synthesis Methods

SPSS program version 29 (IBM Corp., Armonk, NY) was utilized to perform a meta-analysis determining the mean weight loss across patients who underwent beinaglutide therapy. Mean differences were used for effect sizes as the outcome measures were consistent. If studies reported multiple time points or dose escalations, the longest follow-up and final maintenance dosages were used to maximize comparability across studies. Given the substantial heterogeneity between patients with and without T2DM, we performed separate meta-analyses for each group. This provides more accurate pooled effect estimates and reduces the statistical and clinical heterogeneity from pooling these populations. Heterogeneity was calculated using Cochran’s Q, Higgins’ I-squared, and Tau squared. The fixed or random-effects models were chosen based on the level of heterogeneity. Forest plots were created with GraphPad Prism version 10. Given the limited amount of studies, no subgroup analyses or meta-regression were performed.

Results

**Figure 1:**
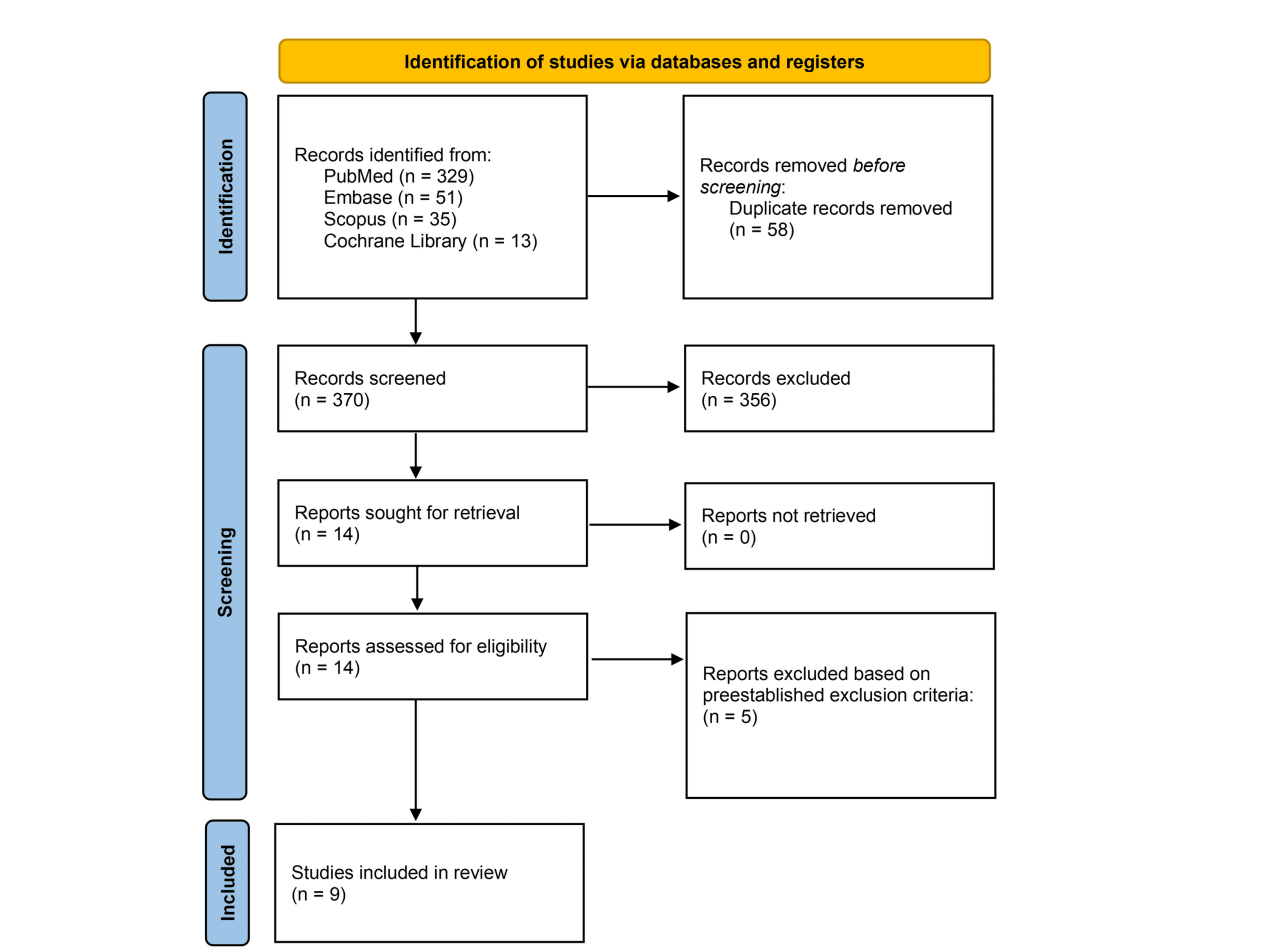
PRISMA flow diagram of the article selection process

The initial search yielded a total of 428 studies from PubMed, Scopus, Embase, and Cochrane Library. After removing 58 duplicate articles, the remaining 370 articles were screened based on their title and abstracts, leaving 14 to be reviewed. A thorough full-text review was conducted and yielded nine studies to be included in this systematic review [9,10,13-19]. The screening process can be visualized in Figure 1.

Demographic Data

Across all nine studies, there were a total of 1,268 (602 diabetic and 666 non-diabetic) patients who completed the studies. Of these, 747 patients were assigned to beinaglutide and 502 to comparator therapy. The mean age was 41.7, ranging between 25.4 to 53.3 years (Table 1).

**Table 1:** Patient demographics

Author	Number of Patients	Sex (Female, Male)	Mean Age in Years (SD)	Diagnosis	Concurrent Medications
Chen et al., 2024 [13]	420	218, 202	36.10 (8.90)	Non-diabetic	None
Fan et al., 2024 [16]	50	22, 28	50.00 (14.10)	Diabetic	None
Gao et al., 2022 [14]	78	34, 30	32.40 (1.50)	Non-diabetic	None
Han et al., 2023 [17]	134	Not reported	51.95 (8.75)	Diabetic	Metformin
Liu et al., 2023 [9]	68	19, 49	52.95 (19.68)	Diabetic	None
Wang et al., 2021 [18]	36	12, 24	46.25 (13.55)	Diabetic	None
Wen et al., 2023 [15]	64	64, 0	26.09 (3.78)	Non-diabetic	None
Wu et al., 2022 [19]	104	43, 61	31.67 (7.33)	Non-diabetic	Anti-hypertensive (19), lipid-lowering (5), uric acid-lowering (3), liver protection (2)
Zhang et al., 2019 [10]	314	151, 163	47.6 (10.5)	Diabetic	Insulin glargine (96.08), metformin (1.88), insulin glargine + metformin (0.94), anti-hypertensive (136.89), lipid-lowering (82.85)

Study Characteristics

The nine included studies encompassed six RCTs, two prospective cohort studies, and one retrospective cohort study, comparing beinaglutide with other therapies such as metformin, insulin glargine, lifestyle modifications, and other standard therapies. The mean follow-up was 39.7 weeks, ranging from 12 to 168 weeks (Table 2).

**Table 2:** Study characteristics SQ: Subcutaneous; TID: Three times per day; BID: Twice per day; iGlar: Insulin glargine.

Author	Number of Patients Assigned to Beinaglutide	Beinaglutide Intervention	Number of Patients Assigned to Comparator	Comparator Intervention	Number of Patients Assigned to Placebo	Mean Follow-Up (Weeks)
Chen et al., 2024 [13]	282	0.2 mg, SQ, TID	None	None	138	16
Fan et al., 2024 [16]	25	0.1 mg, SQ, TID	25	Lifestyle modification	None	24
Gao et al., 2022 [14]	32	0.2 mg, SQ TID	32	0.5 g metformin orally, TID	None	12
Han et al., 2023 [17]	67	0.1 mg, TID	67	Aspart 30 combined with metformin	None	168
Liu et al., 2023 [9]	35	Week 1: 0.1 mg, TID After: 0.2 mg, TID	33	iGlar: starting dose of 0.2 IU/kg or 10–12 IU per day, SQ, before bed. At the end of 8 weeks: enrollees who reached optimal glucose control remained on their current treatment; otherwise, they were administered a combination of beinaglutide and iGlar.	None	16
Wang et al., 2021 [18]	36	BID, before meals. Starting dose: 0.06 mg each week. Goal: 0.1 mg.	None	None	None	13
Wen et al., 2023 [15]	30	SQ, combined with oral metformin (850 mg), BID starting dose: 0.1 mg, TID 2 weeks later: 0.2 mg, TID	30	Metformin: 850 mg, BID	None	12
Wu et al., 2022 [19]	25	0.2 mg, SQ, BID	Lifestyle modification: 57. Multiphase modified ketogenic diet: 22	Lifestyle modification: hypocaloric balanced diet, multiphase modified ketogenic diet: 2 cycles of multiphase diet	None	12
Zhang et al., 2019 [10]	215	Baseline groups (daily): 0.2 mg, 0.3 mg, 0.4 mg, 0.45 mg, 0.6 mg. Dose escalation (1–2 weeks): 0.2 mg, 0.3 mg, 0.4 mg, 0.42 mg, 0.48 mg, and 0.6 mg	96	Beinaglutide dosage combined with iGlar	None	84

Beinaglutide dosage protocols varied across studies and are shown in Table 2. Among these trials, four investigated the efficacy of a 0.6 mg daily dosage of beinaglutide compared to placebo, metformin, and insulin glargine [9,13-15]. In two other trials, the comparison involved 0.3 mg of beinaglutide daily versus lifestyle modifications or insulin aspart 30 combined with metformin [16,17]. Additionally, one trial compared patients who received a baseline dose ranging from 0.2 to 0.6 mg, followed by individual escalation combined with insulin glargine [10]. Two trials did not specify comparator dosing information [10,17]. Additional heterogeneity with dietary and lifestyle modifications was also observed [18,19].

Study Quality and Risk of Bias Assessment

To assess the study quality and the risk of biases present within the studies, the MINORS and Cochrane Risk of Bias criteria were used, given the inclusion of both RCT and non-RCT studies [11,12]. The reviewed studies had a MINORS score that ranged between 19 and 23 for comparative studies [9,10,13-19]. The overall risk of bias was low, and the full breakdown is shown in Table 3.

**Table 3:** Methodological quality and risk of bias

Author	Clearly Stated Aim	Inclusion of Consecutive Patients	Prospective Data Collection	Endpoints Appropriate to Study Aim	Unbiased Assessment of Study Endpoint	Follow-Up Period Appropriate to Study Aim	Loss to Follow-Up Less than 5%	Prospective Calculation of Study Size	Adequate Control Group	Contemporary Groups	Baseline Equivalence of Groups	Adequate Statistical Analyses	Total Score
Chen et al., 2024 [13]	2	2	2	2	2	1	1	2	2	2	2	2	22/24
Fan et al., 2024 [16]	2	2	2	2	2	1	0	0	2	2	2	2	19/24
Gao et al., 2022 [14]	2	2	2	2	2	1	1	0	2	2	2	2	20/24
Han et al., 2023 [17]	2	2	2	2	2	2	1	2	2	2	2	2	23/24
Liu et al., 2023 [9]	2	2	2	2	2	1	1	2	1	2	2	2	21/24
Wang et al., 2021 [18]	2	2	2	2	2	2	2	0					14/16
Wen et al., 2023 [15]	2	2	2	2	2	1	1	2	2	2	2	2	22/24
Wu et al., 2022 [19]	2	2	2	2	2	1	1	0	1	2	2	2	19/24
Zhang et al., 2019 [10]	2	2	1	2	2	2	1	0	1	2	2	2	19/24

The Cochrane Risk of Bias tool was also used to assess bias in RCT studies [12]. The sequence generation showed a low risk of bias across five studies, with one study demonstrating a high bias. Both the allocation concealment and blinding of outcome assessors showed varying risks of bias. The blinding of participants and personnel showed a low risk of bias for one study and a high risk of bias for five studies. The risk of bias was low for both the incomplete outcome data and the selective outcome reporting. In terms of other sources of bias, two studies showed a low risk of bias, while four studies were identified to have a high risk of bias. The complete breakdown is shown in Table 4.

**Table 4:** Cochrane risk of bias tool

Author	Sequence Generation	Allocation Concealment	Blinding of Participants and Personnel	Blinding of Outcome Assessors	Incomplete Outcome Data	Selective Outcome Reporting	Other Sources of Bias
Chen et al., 2024 [13]	Low	Low	Low	Low	Low	Low	Low
Fan et al., 2024 [16]	Low	Unclear	High	High	Low	Low	High
Gao et al., 2022 [14]	Low	Unclear	High	High	Low	Low	High
Han et al., 2023 [17]	Low	Low	High	Unclear	Low	Low	Low
Liu et al., 2023 [9]	Low	Unclear	High	High	Low	Low	High
Wang et al., 2021 [18]	High	High	High	High	Low	Low	High

Weight Loss

The mean change in weight following beinaglutide therapy was -6.2% (-1.5 to 12.9) in patients with diabetes (five studies) and -7.1% (-6.0 to -9.7) in patients without diabetes (four studies). Comparators showed a change in weight of -4.6% (-0.7 to -13.3) and -4.5% (-2.4 to -7.9) in patients with and without diabetes, respectively.

**Figure 2:**
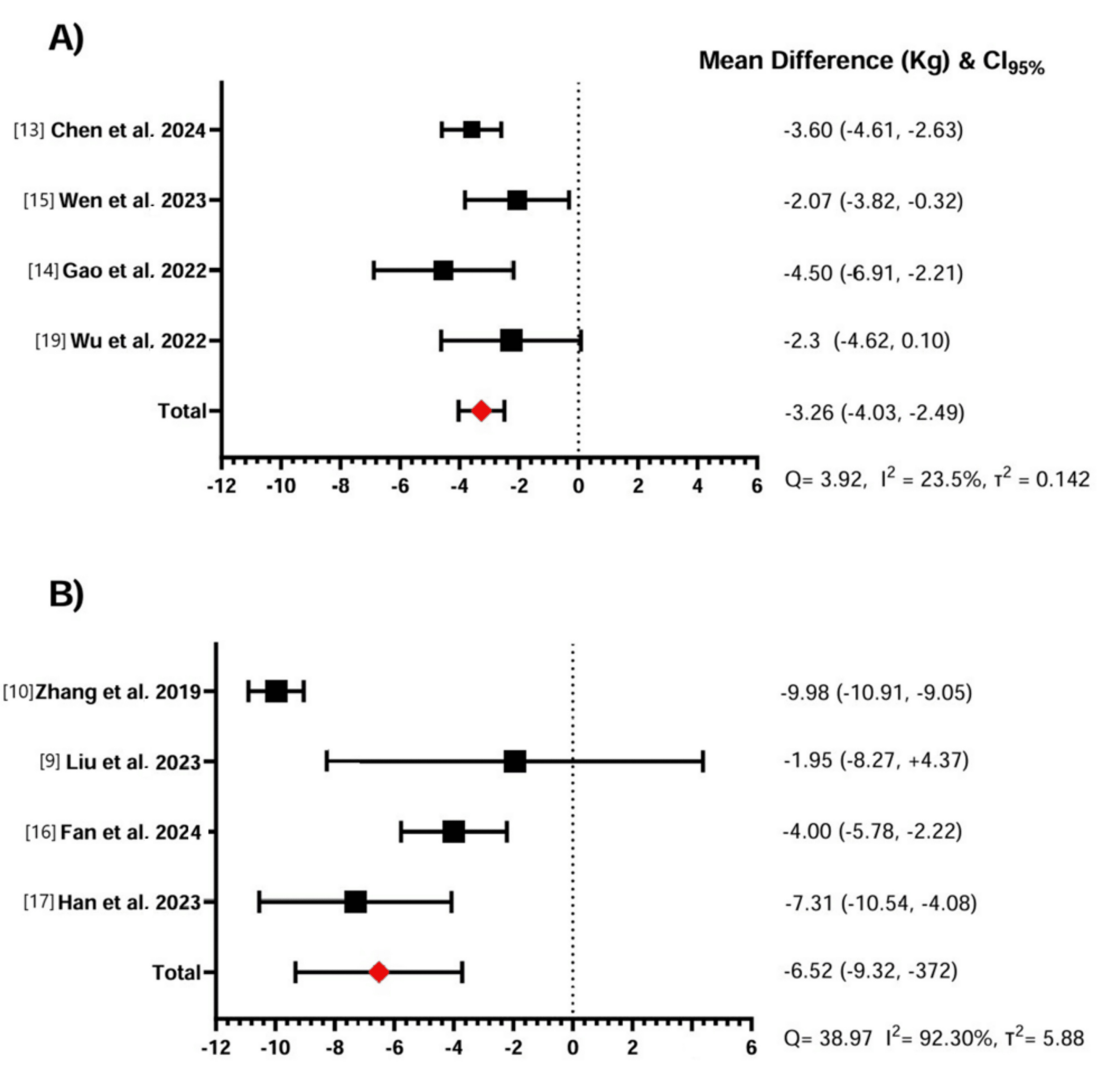
Effect of beinaglutide in body weight reduction in patients with obesity or diabetes Panel A illustrates the mean difference in weight (kg) along with the 95% confidence intervals (95% CI) for patients with obesity. The fixed-effects model was applied due to the low heterogeneity observed in the data (Q = 3.92, I² = 23.5%, τ² = 0.142). The overall effect size indicates a significant reduction in body weight. Panel B displays the effect of beinaglutide on patients with diabetes, where the random-effects model was used due to the high heterogeneity of the included studies (Q = 38.97, I² = 92.30%, τ² = 5.88). The individual study results and the overall effect size suggest notable variations in weight reduction across studies.

Meta-analysis with patients without diabetes (four studies) found a mean weight loss of -3.26 kg (95% CI: -4.03 to -2.49) with a low I2 of 23.5% (Figure 2, Panel A).

The overall pooled effect suggests a statistically significant reduction in body weight. The low heterogeneity of the data indicates consistency among the included studies. The meta-analysis of patients with diabetes (four studies) found a mean weight loss of -6.52 kg (95% CI: -9.32 to -3.72) with a high I^2^ of 92.3% (Figure 2, Panel B). The overall pooled effect suggests a significant reduction in body weight, but with notable variability across studies. A summary of weight loss changes across the individual studies is given in Table 5.

**Table 5:** Effect of beinaglutide on body weight compared to other therapies in patients with diabetes/obesity *Data is reported as mean (95% confidence interval). Comparator is defined as an active comparator with another weight loss therapy.

Author	Pre-beinaglutide Intervention (kg)	Post-beinaglutide Intervention (kg)	% Change (SD)	Pre-comparator Intervention (kg)	Post-comparator Intervention (kg)	% Change (SD)
Diabetes						
Fan et al., 2024 [16]	85.20 (14.9)	79.70	-6.5	87.40 (20.2)	85.90	-1.7
Han et al., 2023 [17]	84.34 (13.86)	79.30 (12.92)	-6.0	83.12 (16.09)	85.39 (15.94)	2.7
Liu et al., 2023 [9]	69.73 (14.45)	8 weeks: 67.67 (14.49); 16 weeks: 68.21 (15.56)	8 weeks: −2.06 (−2.88, -1.23)*; 16 weeks: −1.52 (−3.91, −1.34)*	72.36 (10.57)	8 weeks: 72.50 (10.26); 16 weeks: 71.09 (10.13)	8 weeks: 0.14 (−0.51, 0.79)*; 16 weeks: -0.67 (-1.44, 0.09)*
Wang et al., 2021 [18]	88.97 (11.32)	85.18 (11.45)	-4.3	None	None	None
Zhang et al., 2019 [10]	77.63 (11.19)	67.65 (10.75)	-12.9	77.49 (8.28)	67.19 (8.10)	-13.3
Obesity						
Chen et al., 2023 [13]	89.35 (4.6)	83.99	−6.0 (0.4)	88.04 (18.1)	85.93	−2.4 (0.5)
Gao et al., 2022 [14]	94.00 (2.5)	84.90 (2.2)	-9.7	88.00 (2.5)	83.40 (2.4)	-5.2
Wen et al., 2023 [15]	72.97 (6.79)	68.43 (5.92)	-6.2	72.56 (5.93)	70.09 (3.84)	-3.6
Wu et al., 2022 [19]	91.90 (20.0)	86.10 (17.2)	-6.3	Lifestyle modification: 88.20 (13.2). Multiphase modified ketogenic diet: 88.10 (16.2)	Lifestyle modification: 85.00 (13.8). Multiphase modified ketogenic diet: 81.10 (15.5)	Lifestyle modification: -3.6. Multiphase modified ketogenic diet: -7.9

Adverse Events

AEs were observed in 382/404 (94.6%) participants assigned to beinaglutide treatment reported in five of nine studies. Some study participants were unable to tolerate these events, which resulted in the discontinuation of beinaglutide treatment for 51 patients across four studies (9.0%, n = 564) compared to four patients assigned to different agents (2.0%, n = 203). Zhang et al. did not report total AEs, but specific AEs are reported in Table 6. Fan et al., Han et al., and Wang et al. did not report complications [16-18].

**Table 6:** Common adverse events compared to other therapies Data is reported as n (%).

	Beinaglutide						Active Comparator					
	Liu et al., 2023 [9]	Zhang et al., 2019 [10]	Chen et al., 2024 [13]	Gao et al., 2022 [14]	Wen et al., 2023 [15]	Wu et al., 2022 [19]	Liu et al., 2023 [9]	Zhang et al., 2019 [10]	Chen et al., 2023 [13]	Gao et al., 2022 [14]	Wen et al., 2023 [15]	Wu et al., 2022 [19]
Total adverse events	29 (45.7)	Not reported	240 (83.9)	47 (67.1)	47 (58.8)	19 (76.0)	26 (54.5)	Not reported	112 (79.4)	23 (32.9)	33 (41.3)	Lifestyle modification: 10 (17.5). Multiphase modified ketogenic diet: 2 (9.1)
Dizziness	Not reported	54 (17.2)	61 (21.3)	13 (33.3)	Not reported	6 (24.0)	Not reported	Not reported	5 (3.5)	2 (5.1)	Not reported	Lifestyle modification: 1 (1.8). Multiphase modified ketogenic diet: 2 (9.1)
Nausea	9 (25.7)	160 (51)	141 (49.3)	23 (59.0)	8 (25)	16 (64.0)	2 (6.1)	Not reported	10 (7.1)	2 (5.1)	13 (40)	Lifestyle modification: 0. Multiphase modified ketogenic diet: 0
Hypoglycemia	5 (14.3)	Not reported	Not reported	0	Not reported	3 (12.0)	9 (27.3)	Not reported	Not reported	0	Not reported	Lifestyle modification: 0. Multiphase modified ketogenic diet: 0
Vomiting	Not reported	57 (18.2)	60 (21.0)	8 (20.5)	7 (21)	4 (16.0)	Not reported	Not reported	0	0	2 (32)	Lifestyle modification: 0. Multiphase modified ketogenic diet: 0
Diarrhea	Not reported		16 (5.6)	0	0	Not reported	Not reported	Not reported	12 (8.5)	17 (43.6)	8 (25)	Not reported
Headache	Not reported	26 (8.3)	31 (10.8)	0	3 (9)	Not reported	Not reported	Not reported	6 (4.3)	0	0	Not reported
Upper respiratory tract infection	Not reported	Not reported	27 (9.4)	Not reported	Not reported	4 (16.0)	Not reported	Not reported	14 (9.9)	Not reported	Not reported	Lifestyle modification: 3 (5.3). Multiphase modified ketogenic diet: 0
Fatigue	Not reported	Not reported	16 (5.6)	2 (5.1)	1 (3)	2 (8.0)	Not reported	Not reported	3 (2.1)	1 (2.6)	0	Lifestyle modification: 0. Multiphase modified ketogenic diet: 0
Discontinuations due to adverse event (AE)	4 (11.4)	26 (12.1)	17 (5.9)	4 (12.5)	Not reported	Not reported	1 (3.0)	Not reported	1 (0.7)	2 (6.3)	Not reported	Lifestyle modification: 0. Multiphase modified ketogenic diet: 0.

Discussion

This systematic review analyzed nine studies (six RCTs, two prospective cohorts, and one retrospective cohort) evaluating the effect of beinaglutide on weight loss, including a total of 1,268 patients (747 beinaglutide/502 comparators) with a mean follow-up of 47.6 weeks. Five studies focused on patients with diabetes (602 patients), and four on patients without diabetes (666 patients). The main findings indicate that beinaglutide produced higher percentages of weight loss in patients with and without diabetes compared to their respective comparators. Among the five studies reporting complications, a high rate of AEs was observed among beinaglutide patients at 382 (94.6%), mainly consisting of minor and moderate GI AEs. Overall, the safety profile of beinaglutide is similar to the findings for other GLP-1 RAs (semaglutide, liraglutide, and tirzepatide) [20].

Beinaglutide is a short-acting recombinant GLP-1 RA that shares a 100% similar amino acid sequence to human GLP-1. In mouse pancreatic islets, it has been shown that beinaglutide stimulates GLP-1 R-dependent cyclic AMP (cAMP) and enhances glucose-dependent insulin release in the HEK 293 cell [8]. Additionally, the time to peak concentration (Tmax) is about five minutes, which is close to the half-life of endogenous GLP-1 of 1.5 to five minutes [8]. Similar to other GLP-1 RAs such as semaglutide and liraglutide, the reduction in body weight is mediated by decreased gastric emptying and reduced food intake via binding to central receptors in the hypothalamus, hindbrain, hippocampus, and mesolimbic circuitry. Weight loss has also been attributed to brown adipose tissue (BAT) thermogenesis, mediated by GLP-1 receptors in the ventromedial or dorsomedial nuclei of the hypothalamus. In mice models, beinaglutide did see an increased expression of white adipose tissue (WAT) and BAT, but without an increase in basal metabolic rate. Therefore, the authors state that beinaglutide’s physiological effects are mainly mediated by suppressed food intake and decreased lipid metabolism instead of WAT and BAT. Another proposed mechanism of beinaglutide’s weight loss effect is via the normalization of gut microbiota by increasing the levels of *Faecalibacterium prausnitzii*. Specifically, a negative relationship was found between *F. prausnitzii* and T2DM patients, thus beinaglutide’s glycemic and weight control is suggested partly due to its ability to alter gut microbiota [21]. Beinaglutide does not appear to produce similar levels of weight loss as compared to semaglutide (14.9% to 17.4%) or tirzepatide (16.5% to 22.4%) [22,23].

Obesity causes several notable consequences, including chronic inflammation and reactive oxygen species that lead to insulin resistance via impaired insulin receptor signaling pathways. GLP-1 RAs, besides their ability to increase cAMP levels, are also able to reduce oxidative stress by increasing nuclear factor erythroid 2-related factor 2 protein expression, which decreases the production of advanced glycation end products. Thus, this leads to improved insulin sensitivity [24]. Another possible indication is for polycystic ovary syndrome (PCOS) patients, as weight control is one of the mainstays of treatment for this condition to lower metabolic risk factors and restore reproductive ability. Similarly, GLP-1 RAs can also be a benefit for nonalcoholic steatohepatitis (NASH) patients, given that obesity and T2DM are major risk factors for the development of this condition. GLP-1 RAs can affect lipid metabolism by regulating preadipocyte differentiation and modulating lipid metabolism (decreasing lipogenesis and increasing lipolysis) [25]. In ob/ob mice, it has been shown that beinaglutide affects the composition of glycerolipids, phosphoglycerides, sphingolipids, and lipid metabolism gene expression (β-oxidation (Ppara, Acadl, and Acox1), mitochondrial function (Mfn1 and Mfn2), antioxidation (Sod2), Sirt 1) [26]. However, the underlying mechanism of action on adipocyte tissue is still unclear [8]. The reduction in body weight provided by GLP-1 RAs is correlated with decreased levels of liver damage markers and hepatic fat, thus promoting the resolution of steatohepatitis and other NASH parameters [26].

GLP-1 RAs are traditionally developed for T2DM patients but have also been recently approved for weight loss in patients with obesity (e.g., liraglutide in 2014, semaglutide in 2021, and tirzepatide in 2023) [27]. However, these are recommended as adjuncts to lifestyle changes, including a reduced-calorie diet and increased physical activity. An additional indication for semaglutide (Wegovy, 2.4 mg) that was approved by the FDA​​​​​​​ in 2024 is to reduce the risk of cardiovascular disease [28]. Additionally, dual (GLP-1/glucagon receptor) and triple (GLP-1/glucagon/gastric inhibitory peptide receptor) agonists are also being investigated, and future research is needed to quantify their effects compared to GLP-1 RAs. However, semaglutide and tirzepatide both come with high costs, often exceeding $1000 a month [29,30]. These medications are often prescribed for long-term weight management, leading to additional overall costs. Globally, there is a huge push for the development of alternative GLP-1 RAs, notably in China and India, where a large portion of global obesity or overweight prevalence can be found [30]. These biosimilar compounds are designed as cheaper versions of branded drugs but are derived by modifying living organisms (e.g., yeast) [31]. The tremendous healthcare burden of diabetes and obesity worldwide has led to increased development of biosimilar products, as they can lower health costs and increase access to medications if similar clinical outcomes are observed. A recent comparison between biosimilar liraglutide and reference liraglutide demonstrated similar efficacy and safety profiles [31]. Although beinaglutide is not yet approved by the FDA​​​​​​​ in the United States, preliminary clinical trials conducted in China, reviewed in this paper, presented promising results for its use in weight loss. Beinaglutide can effectively promote weight loss in both patients with and without diabetes, with greater reductions observed in body weight compared to control groups.

Limitations

Despite these promising findings for beinaglutide, these results must also be evaluated within the context of its limitations. First, all of these studies were conducted in one ethnic background and may not apply to patients from different areas of the world. Future studies should focus on conducting multi-ethnic and multi-center studies across the globe. Second, there were no direct comparative head-to-head trials with other GLP-1 RAs, thus preventing a concrete statement of superiority or inferiority against others in its class. Longer-term studies are also required to better clarify the long-term weight loss effects of beinaglutide. Furthermore, the comparative effectiveness of beinaglutide against other therapies such as metformin, insulin glargine, and lifestyle modifications varied across studies. Most studies reported superior weight loss results associated with beinaglutide, but one study reported equal weight loss outcomes when compared to lifestyle modification with a ketogenic diet. In some instances, beinaglutide demonstrated superior weight loss outcomes, while in others, it was comparable or less effective. These findings underscore the need for direct comparative studies to establish its relative efficacy and safety. Studies involving diverse patient populations and direct comparisons with other GLP-1 RAs are crucial to determine its potential advantages and establish its role in weight loss management. Third, although there were six RCTs in this study, the other three were cohort studies, thus possibly introducing bias that can affect the results analyzed. Fourth, the literature search did not include gray literature, clinical trial registries, or non-English-language databases. Although the included studies were conducted in China, these were published in English-language journals and indexed in international databases. However, the exclusion of these sources may still introduce language and publication bias.

## Conclusions

Beinaglutide demonstrates greater weight loss compared to placebo or active comparators, but with a greater amount of AEs, consisting mainly of minor to moderate gastrointestinal AEs. However, these preliminary findings require further comparative and long-term studies to fully understand their efficacy, safety, and potential benefits over existing therapies. Beinaglutide and other compounds will continue to be developed in hopes of providing more affordable access and similar efficacy as currently available GLP-1 RAs.

## Appendices

Appendix 1: Search query across all databases

PubMed: (beinaglutide) AND (weight loss OR outcomes OR efficacy OR diabetes OR diabetic OR overweight OR obesity)

Embase: (beinaglutide) AND (weight loss OR outcomes OR efficacy OR diabetes OR diabetic OR overweight OR obesity)

Scopus: (beinaglutide) AND (weight loss OR outcomes OR efficacy OR diabetes OR diabetic OR overweight OR obesity)

Cochrane Library: (beinaglutide) AND (weight loss OR outcomes OR efficacy OR diabetes OR diabetic OR overweight OR obesity)
